# Project TEACH School-Focused Consultation and Community Collaboration: A Multidisciplinary Pilot Intervention to Reduce Mental Health Disparities in Upstate, NY

**DOI:** 10.3390/healthcare14091194

**Published:** 2026-04-29

**Authors:** Nayla M. Khoury, Maureen Ryan, Jessica Hoff, Melissa Dhundale, Eric MacMaster, Ryan D. Heath

**Affiliations:** 1 SUNY Upstate Norton School of Medicine, Syracuse, NY 13210, USA; 2 TST BOCES (Student Health Systems), Ithaca, NY 14850, USA; 3 School of Social Work, Syracuse University, Syracuse, NY 13244, USA

**Keywords:** school-focused collaborative care, multidisciplinary teams, school mental health, mental health disparities

## Abstract

Background: Youth mental health needs are critically undertreated. Access and engagement remain challenging, particularly for disadvantaged youth, due to barriers such as limited clinic hours, insurance, transportation, bias, and stigma. School-focused collaborative approaches may reduce mental health care inequities. In this study, we illustrate a pilot intervention by process documentation, participant feedback, and two case studies. Method: To address local service gaps, a virtual collaborative care process was piloted with a child and adolescent psychiatrist (CAP) and psychologist from Project TEACH, a New York State Office of Mental Health funded Child Psychiatry Access Program (CPAP), primary care representatives, and multiple school mental health teams. Demographic data, participant feedback and the collaborative process is reviewed with two case studies created to highlight the collaborative process. Results: Most participants report utility and felt supported. The majority also report a positive impact on communication and collaboration between teams serving students; challenges with family follow up persist. Streamlined communication and consent was helpful. Demographic data suggests that this model can help reach disadvantaged youth. Conclusions: School-focused collaborative mental health requires regular communication and coordination between youth-serving providers. This pilot implementation study shows promise for reaching disadvantaged youth and providing multidisciplinary support.

## 1. Introduction

Professional bodies including American Academy of Child And Adolescent Psychiatry (AACAP), American Academy of Pediatrics (AAP) and Children’s Hospital Association (CHA) have called for integrated mental health care in primary care and school-based services to support youth wellbeing to meet growing mental health needs [1]. Both modalities demonstrate promise in improving youth outcomes, although there are few studies reviewing their use together. This pilot study evaluates the process and preliminary results of a program that combines elements of integrated collaborative care and school-based care to improve outcomes for youth in need. Integrated and collaborative care has been well-established as models to address mental health shortages nationally. Collaborative mental health care, defined by the American Psychiatric Association as a team led by a primary care provider and including behavioral health specialists such as psychiatrists, other mental health professions, and a behavioral health care manager can provide access to youth who may otherwise not seek psychiatric care [2]. Evidence supports the ability of integrated primary care interventions to improve treatment outcomes in youth [3,4]. This study adds to the literature by reviewing and evaluating the process of one creative school-focused collaborative intervention to address youth mental health needs in school settings.

### 1.1. Mental Health Challenges of School-Aged Children

Youth mental health has been in an ongoing crisis since the COVID19 pandemic exacerbated existing gaps, with school-aged youth experiencing higher rates of depression, anxiety, behavioral issues, and suicidal behaviors [5]. The February 2023 JAACAP Clinical Update highlights that 1 in every 4–5 youth in the United States may be affected by a mental health disorder. Specifically, over fifty percent of primary care encounters address mental health concerns and primary care providers prescribe most psychotropic medications for youth in the United States, although more than half may go untreated [6]. The timeline of these conditions makes them some of the costliest pediatric conditions, both in direct healthcare costs as well as the costs of long-term societal impact. Another challenge includes the overall shortage of mental health providers, especially child and adolescent psychiatrists. This is experienced even more severely in rural areas and those areas with higher socioeconomic marginalization. A troubling result of this resource scarcity for youth with mental health concerns is high variability in care quality and delivery, often delaying appropriate diagnosis and treatment [6].

### 1.2. Obstacles to Accessing Care & Follow Up

Less than half of youth diagnosed with a mental health disorder are estimated to receive treatment [6]. For disadvantaged and minority youth, mental health disparities are even more stark. A recent cross-sectional study among adolescents in the US finds that less than one quarter of non-Hispanic black adolescents and approximately one quarter of Hispanic adolescents receive mental health treatment compared to one third of non-Hispanic white youth [7]. Such disparities are perpetuated by insufficient providers, restrictive clinic hours, insurance barriers, transportation, and stigma [8]. The consequences of unmet needs include disruptive behaviors, hospital visits, suicide attempts and challenges with transitioning into adulthood [9,10].

### 1.3. Disparities and School-Based Collaborative Solutions

Through the utilization of school-based collaborative care, a model of collaboration that includes school-based mental health services in addition to primary care, access for youth can be further improved as schools are in a unique position to identify youth in need and connect families to mental health services. Such models can improve behavioral health accessibility and improve the capacity of schools to provide behavioral health services [11,12]. Given ongoing issues as described above, which create both obstacles to accessing care and difficulties maintaining care/avoiding attrition, creative collaborative solutions are needed to address persistent shortage gaps. One recent study from 2025 reviewed a school-focused collaborative consultation model; finding that via virtual education/consultation sessions, providers in SBHCs can improve their ability to address mental health service shortages and reduce youth experiencing crises. These improvements were rendered most effective when providing tangible resources, case-based consultations with child psychiatrists, and skills-based learning [13]. Realtime benefits of this model, especially in an area like Ithaca, reduce long wait times for the few child and adolescent mental health providers, reduce excessive commutes/transportation barriers, and encapsulate mental health as part of primary care. All of these play an important role in the access and retention of children and adolescents.

The 2022 National Census of School-Based Health Centers (SBHCs) highlighted that SBHCs can provide primary care services in school settings and increase access for youth identifying as black, indigenous and people of color [14]. Furthermore, that same report also highlighted SBHCs as having a unique ability to address other disparities, including, but not limited to food security, facilitating access to interpersonal relationship and safety services, immigration or acculturation support, transportation-related neighborhood and built environment challenges, and support students accessing academic support services [14]. A study of 5396 youth at 14 SBHC sites in California found that youth from racial/ethnic minorities accessed MH at SBHC at similar levels as white youth and that gender-diverse and LGBTQ+ youth also accessed care at higher or similar rates, respectively, as their same sex and heterosexual peers [15]. Many of these social determinants of health present real-life situations faced by students in Upstate New York. Although SBHC mental health programs are expanding and filling an increasingly necessary role in the identification and treatment of youth mental health, especially those with public or no health insurance coverage, they remain less supported by existing funding and infrastructure [16]. In New York state, only 19% of SBHC are in rural areas and of that group approximately half of those clinics are run by one regional hospital system [17]. The composition of the catchment area of the Ithaca City School District can be broadly described as rural, although there are striking differences between the access to services meant to minimize healthcare disparities and increase support amongst those who live in the more urban core of Ithaca versus those spread out in the far more rural surrounds. Ithaca’s programming in many ways mirrors the rest of the United States, where nationally, although most schools offered some mental health service in 2024–2025, most schools remained ill equipped [5].

Creative collaborative solutions are thus needed to address persistent shortage gaps, and early research has suggested feasibility in nearby areas, but that costs, institutional differences and data sharing remain barriers [18]. A recent study reviewing school-focused collaborative consultation model suggested that virtual education/consultation sessions can be used in SBHCs to help address mental health service shortages and reduce youth experiencing crises [13]. While evidence for SBCH is growing, hybrid school-based collaborative care models including both school and primary care teams have not been a focus of extensive study to date, in spite of a call for increased implementation and study of school-based collaborative care [11,19,20]. To help address this gap, this implementation study documents and evaluates the feasibility and acceptability of one school-focused collaborative pilot program to address growing youth mental health needs in school settings.

## 2. Materials and Methods

### 2.1. Setting

Ithaca City School District (ICSD) and the approximately 5000 students served provided an opportunity to develop a creative collaborative solution to persistent mental health shortage gaps in a rural setting. To do so, the district utilized two external partnerships: (1) Project TEACH, New York State’s Office of Mental Health Child Psychiatric Access Program (CPAP) and (2) Thompson County-Tioga (TST) Board of Cooperative Educational Services (BOCES; in New York State, BOCES are cross-district educational partnerships). Together, ICSD, Project Teach and the TST BOCES medical team develop a model of interprofessional collaboration across systems of care. A weekly, hour long virtual collaborative process referred to as a virtual huddle that included Project TEACH child adolescent psychiatrist, fellows and psychologist; regional district medical director team (TST BOCES medical director team); primary care office providers (PCPs); school staff (school psychologists, social workers, teachers, CSE support staff, principals), and ancillary services (case managers, child protective services, community based therapists, etc.) was created to address limited access to psychiatry and need for scaffolding across systems for high risk youth.

### 2.2. Participants

School staff from participating districts in the TST BOCES region or PCP office identified students/patients that they believe would benefit from interprofessional collaboration, obtained parental/guardian consent for the collaboration (FERPA/HIPAA consent), and submitted an online referral to the TST BOCES team. School staff and/or PCP office referred students/patients that were struggling with behaviors such as social behaviors, school attendance, social determinants of health, medication management, sexualized behaviors, suicidal or homicidal thoughts. Inclusion criteria included any student in the participating districts (K through 12th grade) with a current PCP; there were no exclusion criteria. General Ithaca population information regarding race and ethnicity for the time studied included a population pool of 63% White-identifying students, 7% Black-Identifying, 9% multi-racial identifying and 9% Hispanic-identifying. The TST-BOCES School Health team consists of a Board-Certified Pediatrician and a Board-Certified Physician Assistant, each with over 20 years of experience taking care of children with mental health needs and who were working towards completing a 40-week intensive Psychiatric Education for Pediatric Providers (PEPP) training. Many students/patients will require only a call to a PCP making them aware of an attendance issue, or a social determinant of health barrier, allowing for further medical office interventions.

### 2.3. Procedures

Based on information received from schools/PCPs and gleaned from the mental health screening portion of the Health Assessments, the TST BOCES team triaged and tiered referrals and determined mental health community connections to initiate in support of the student.

Many students/patients require only a call to a PCP making them aware of an attendance issue, or a social determinant of health barrier, allowing for further medical office interventions. Cases with complex mental health needs (such as an under-managed or medication resistant mental health diagnosis, multiple psychiatric diagnoses, unidentified diagnoses), active engagement with a PCP, and demonstrating a need for additional school support met criteria for the Project TEACH tier huddle. Additional consents are obtained for the virtual interprofessional meeting and school staff, PCPs, and involved community partners are invited to the HIPAA approved Project TEACH and TST BOCES standing meeting virtual platform. The huddle is moderated by the TST BOCES team and begins with the referring school team providing a brief synopsis of the primary concern, current resources/school accommodations, and barriers to care. The remainder of the hour is driven by Project TEACH specialists clarifying questions leading to the discussion, resource/medication suggestions and sharing of information between systems of care. Project TEACH provides post huddle support, which can include: a one-time extended psychiatric evaluation at no cost as part of the CPAP grant; ongoing direct collaboration with the PCP regarding medication management and other treatment recommendations; or a re-huddle to assess outcome of recommended school and community based interventions and identification of any additional needs. Similarly, the TST BOCES team continue to be a resource for both the school and PCP providing continuing support and collaboration after the huddle. All participants of the meeting are sent a follow-up email with a synopsis of the meeting and action plan(s) for each team. This process has been iterative and evolved based on feedback since its inception.

SUNY Upstate Institutional Review Board (IRB) was consulted and deemed this study exempt from review based on criteria.

### 2.4. Measures

The current study examined the implementation of a school-based collaborative care model across multiple school districts in Upstate New York. This study was a preliminary implementation study [21], investigating the feasibility and acceptability of a newly developed school-based collaborative care model. The study included (1) narrative summary of program development, completed by key team leaders (the first through fourth authors); (2) student patient characteristics; (3) participant survey descriptive statistics; (4) rapid qualitative analysis of participant feedback.

To develop a narrative summary of program development, team members involved in the development reviewed notes and administrative materials describing program development. As the school-focused consultation and community collaboration process evolved, the first four authors met and completed iterative revisions to streamline coordination. A tiered process was adapted from collaborative care models to the school-based setting and is described below.

Demographic information from youth who were reviewed in the collaborative care huddle tier was collected, including common chief complaints, age, grade, race, ethnicity, and case complexity as well as family function. Case complexity ranged from simple (straightforward, basics of medication management), to somewhat complex (multiple diagnostic possibilities) to complex (complicated family or social situation, 3 or more failed medical trials), to very complex (difficult interactions, office management of imminent suicidality/physical aggression, questions of CPS involvement), determined by the CAP at time of consultation. Family function descriptors ranged from healthy (supportive, caring, able to assist child function independently), to mild-moderate dysfunction (family or parent-child conflict significant factor in child’s life, although individually parent/caretaker able to provide at least some support) to severe dysfunction (family or parent-child conflict major factor impacting child’s life) to extreme dysfunction (unable to provide for basic physical, psychological, medical needs with CPS involvement), determined by CAP at time of consultation. All elements of demographics are recorded into a de-identified CPAP system by CAPs involved in the collaborative process.

Preliminary feedback was obtained from collaborative participants during the first year of implementation (2023–2024) and additional feedback from collaborative participants obtained from the 2024–2025 academic year by solicitations over email and via Survey Monkey. Data was collected in aggregate and anonymously, and themes identified, broken into the categories of impact and challenges. Two specific cases were chosen to highlight the complexity of youth served and the processes which led to improved treatment access.

To analyze qualitative data, rapid qualitative analysis (RQA) was used. RQA is a method used to identify key themes in applied work, especially when timely findings are necessary to guide implementation and design [22] and has been effectively utilized in behavioral health [21] and implementation research [23]. Consistent with RQA principles, data was collection prioritized high-value informants, followed by analysis that identification of themes from qualitative survey responses and administrative materials were organized into themes by the first author, and reviewed by the second and fourth authors. Consensus in themes and interpretations were refined and finalized via group discussion. Preliminary results were then brought back to school district and agency staff for member-checking to ensure accuracy in interpretation. Lastly, two illustrative cases were chosen to highlight the complexity of youth served and the processes which led to improved treatment access (details were edited to maintain patient confidentiality).

## 3. Results

### 3.1. Narrative Summary of Process Development

The school-focused consultation and community collaboration grew out of Project TEACH, a New York State funded Child Psychiatric Access Program (CPAP) that has been around for over 10 years [24]. In 2022, child and adolescent psychiatrists (CAPs) partnered with school districts to create a virtual collaborative care process to support school-based teams and youth with mental health needs. This partnership included medical representatives from primary care offices, school staff such as school psychologists, social workers, teachers, CSE support staff and principals as well as the opportunity of child and adolescent psychiatry fellows to participate in a case consultation model.

Utilizing Project TEACH call consultation time and education time for a CAP fellow, as well as a grant obtained by the TST-BOCES team, consisting of 30 min every week was set aside originally to conduct a virtual huddle with the school and primary care-based teams. Over time, the process for collaborative case consultations with schools became more streamlined, expanding to 45–60 min, including a one-page write-up to organize the schools’ concerns. In addition, a consent form was created for parents that describe the collaborative consultation process. Lastly, follow up notes and discussion led by the BOCES team provided processes to give feedback to both primary care and schools post huddle.

**Figure 1:**
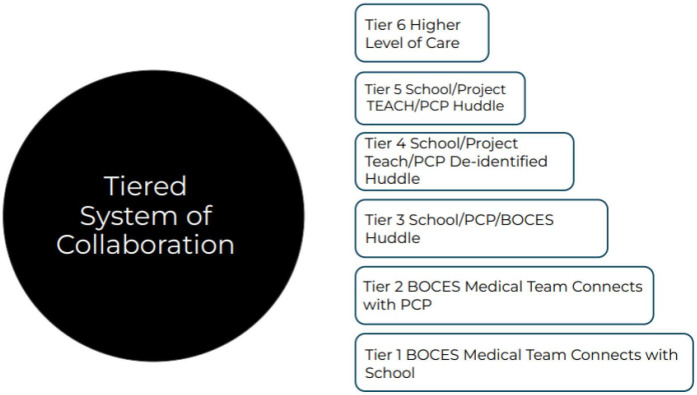
Tiered System of Collaboration.

A tired system of collaboration was developed by the BOCES team (Figure 1) including step 1, connecting with BOCES alone for consultation, up to Tier 5 where the collaborative virtual huddle with Project TEACH was involved. Based on information received from school teams, PCPS, or information gleaned from the mental health screening portion of the Health Assessments, the school health system team triages and tiers referrals and connections. The TST-BOCES School Health team consists of a Board Certified Pediatrician and a Board Certified Physician Assistant, each with over 20 years of experience taking care of children with mental health needs and who were working towards completing a 40-week intensive Psychiatric Education for Pediatric Providers (PEPP) training.

This tiered system has been developed to more effectively connect students to mental health services either within the school or within our community. Tier connections range from direct referrals/connections of PCPs, to school social workers, nurses, psychologists, Racker supported programs such as Possibilities to collaborative meetings with the Syracuse team of Project TEACH to help with a collaborative plan for student mental health support. Presenting youth are initially tiered at a specific level and can change over time.

From Fall 2023–Spring 2025, the BOCES medical team reviewed 269 cases. Of those, 24 were reviewed at Tier 5 collaborative huddle and were all complex cases due to symptom severity, functional impairment and social stressors interfering with access to mental health care. Figure 1 illustrates other tiers of collaboration including Tier 2 with the BOCES team and primary care provider (PCP), Tier 3 with the school, PCP and BOCES team, Tier 4 where educational collaboration occurred with Project TEACH staff de-identified and Tier 5 where an identified huddle occurred, after proper consent was obtained from the identified patient’s family.

Figure 1 depicts a tiered system of collaboration developed with BOCCES Medical Team, school based mental health teams and Project TEACH. Tier 1: The TST-BOCES School Health team connects with a student’s PCP and appropriate school contact to develop the needed coordinated mental health supports. Tier 2: The TST-BOCES School Health Team meets with PCP directly to gather more information for appropriate connections for the child. Tier 3: School/PCP/TST-BOCES School Health Team-facilitated collaboration (i.e., a HUDDLE) to provide support, mental health referrals and next steps for supports such as referrals. Tier 4: Syracuse Project TEACH team/School/PCP/TST-BOCES Team meet to discuss the needs and situation of a de-identified student. Tier 5: Syracuse Project TEACH team/School/PCP/TST-BOCES Team collaborate for next steps, Project TEACH offers One- Time evaluations if needed. Tier 6: TST-BOCES Team connects students with their required higher level of mental health care with Tier 5 being the level where virtual collaborative huddles take place.

### 3.2. Tier 5 Student Patient Characteristics

Table 1 illustrates the demographic and clinical breakdown of patient demographics specifically in Tier 5. Most students were in elementary school with a mean age of 10.6 years. More males than females were discussed in Tier 5 huddle (14 vs. 5). Regarding race, 16% of students discussed in the collaborative huddle were Black-identifying whereas 58% were white race identifying. This is in comparison to the general Ithaca population where approximately 7% are Black-identifying and 63% are White-identifying. In addition, 11% of students in Tier 5 were multi-racial identifying compared with the 9% in the general Ithaca population who identify as multi-racial. 14% of students in Tier 5 were known to be Hispanic identifying compared with the 9% in the general Ithaca population who identify as Hispanic.

Table 2 highlights the complexity of youth discussed in Tier 5 with 82% being identified by CAPs as complex or very complex. In addition, 47% of the students discussed had family functioning identified by CAPs as severe or extreme dysfunction, and 94% had some amount of family dysfunction noted. Most of the cases discussed in Tier 5 went on to have a one-time virtual consultation with Project TEACH and student and family to aid in next step guidance for the primary care and school teams.

### 3.3. Multidisciplinary Participant Feedback

Table 3 shows qualitative feedback from Tier 5 huddle participants, with a response rate of 44%. Invaluable learning and advice was reported by 21% of respondents. One participant noted,

The HUDDLE process has been invaluable. Our teams have gained better understanding of medications and resources to support our students. Many of my colleagues agree that HUDDLE are the most helpful consultations we access for our students.

17% respondents commented on the multidisciplinary collaboration as a strength and being able to understand a student from multiple perspectives. Noted one participant, “It is extremely helpful to be aware of all the providers involved and to be able to share information from school, where we see the majority of the day to day concerns and functional impact.”

14% of respondents commented on having a medical or objective outside lens as helpful to validate concerns by members of the school team. One participant reported, “I think having a medical lens to validate our concerns and help guide the interventions at school was vital. It was difficult parsing what we ought to do to help the student to retain their dignity and what we were potentially covering up and whitewashing by over supporting the student with an unsupportive parent.”

Lastly, improved transitions of care was important to one respondent who noted, “It was a great way to transition care. We received excellent information and knew what to expect and look for”. Challenges identified included paperwork which felt extensive to one participant, and follow up communication between staff as being less than anticipated for 10% respondents, including sharing of documentation as well as follow up for the identified patient family. Stated one participant, “Follow-up is important, especially when recommendations are made for the family to follow through and there is not always a case manager assigned to support the family in follow up appointments or actions, which is difficult for the school to do from our role.”

The majority of huddle participants agreed or strongly agreed with the question “Do you feel the huddle process gave you a more accurate and complete understanding of the child’s mental health condition” (87%) and reported that they agreed or strongly agreed with feeling supported by the huddle process (83%). The majority also reported that they agreed or strongly agreed with the question “Do you feel the huddle process positively impacted your ability to communicate between groups, including follow up conversations outside of the huddle” (77%) while slightly more than half reported that they agreed or strongly agreed with the question “Do you think the huddle process positively impacted the students’/patients’ mental health outcomes?”

Feedback obtained from rapid qualitative analysis informed changes to the collaborative process to include shortened form submissions to reduce paperwork, written summary notes following the multidisciplinary collaborative huddles with specific follow-ups noted and point people identified, particularly around following up with family and updating team on follow through or lack thereof. In addition, re-huddles were introduced more regularly to follow up on patients initially identified who were recommended for a one-time psychiatric consultation. This process allowed reciprocal learning and feedback as well as more coordination between school-based teams and primary care offices, which often implement treatment recommendations.

### 3.4. Case Examples

Two cases based on several real student cases illustrate the severity and diagnostic complexity of youth being managed in primary care settings and with considerable scaffolding by school systems. The collaborative huddle provided rapid access to psychiatric evaluation and development of treatment plans for these two youth, who despite their level of impairment, were not connected to any outpatient mental health services.

Case 1: A 14-year-old male Black-identifying adolescent with a history of unspecified anxiety, depression, selective mutism, and autism spectrum disorder was referred to the huddle due to markedly reduced communication across all settings. Through the collaborative huddle process, concerns were identified regarding the rapidity of the deterioration and history of epilepsy and a traumatic brain injury at 18 months. A medical work-up and urgent Project Teach psychiatric evaluation were recommended. Upon evaluation, the youth demonstrated numerous symptoms of catatonia, including limited movements, waxy flexibility, catalepsy, and alogia. Ongoing collaboration over the course of more than a year was essential in helping this family with multiple socioeconomic disparities access appropriate care, including appropriate primary care services as well as initiation of psychiatric medication. An additional collaborative huddle was held to reorient the school in viewing current lack of communication through the lens of catatonia, rather than exacerbation of selective mutism, and provide appropriate accommodations.

Case 2: A 6 yr old biracial Hispanic adopted male was referred primarily due to daily sexualized behavior towards his peers, teachers, and others in the community. In the collaborative huddle, other areas of impulsivity were identified; including running away at home and disruptive behaviors in the classroom. While a slight reduction in sexualized touching of others had been observed in response to school-based behavioral interventions, the youth began touching and wetting himself. The ensuing case consultation discussion led to a review of biopsychosocial factors contributing to current behaviors including the impact of unknown biologic factors, early adoption, longstanding symptoms that were consistent with ADHD, insomnia, an anxious attachment with his adopted grandparent who was easily triggered by his boundary crossing with her and may have been inadvertently exacerbating his curiosity by her response, and a recent visit with biologic parents that had triggered some regressive behavior such as bedwetting. All of these factors appeared to be contributing to current challenges with limit-setting at home and also at school. The huddle process facilitated evaluation through Child Advocacy Center, consultation with Project Teach problem sexual behavior specialist, and a Project Teach psychiatric evaluation. The combination of intensive and multifaceted interventions, including medication management of previously untreated ADHD and in-service by the Project Teach specialist regarding problem sexual behaviors and behavioral plans, were highly successful. Within a very short period of time, the youth had no occurrences of sexualized behavior at school and home.

## 4. Discussion

Collaborative care and school-based health clinics are being called upon as critical solutions to the current youth mental health crisis. School-based collaborative care models are growing to meet this need with growing evidence for engaging disadvantaged particularly in SBHCs [12,15,25]. However, the lack of capital, staff and resources in many settings means that creative solutions are needed to help meet current gaps. This study adds to growing literature by demonstrating the feasibility, acceptability and perceived utility of a collaborative virtual consultation process that evolved over time into a tiered system approach to multi-system collaboration for youth in school settings with mental health needs.

Like Sielaty and colleagues, this model utilized the infrastructure and expertise of a CPAP. Previous studies have demonstrated how CPAPs such as Project TEACH can improve PCP confidence, train PCPs to feel more comfortable diagnosing and treating youth with mental illness, help with linkage and provide emotional support to PCPs [24,26]. The additional value of collaboration with a school based mental health team is access to a holistic biopsychosocial approach, important context for presenting concerns and the ability of school-based teams to engage in certain interventions in the school setting right away and follow up more closely with families who may need further support. These system collaborations are greatly needed given silos in existing systems of care, even in SBHCs with MH care.

Moreover, our hypothesis was that youth served through this model may be more diverse than youth served by traditional psychiatric systems and thus help close disparity gaps. Indeed, although the sample size is small, a review of the data in the first two years reveals that the students identified and discussed in Tier 5 may be more diverse than the general Ithaca school district, reflecting the potential of these huddles to provide quick access to psychiatric services than is typically available.

Lessons learned include the importance of streamlining consent processes between schools and PCP offices so that communication is easier before crisis emerge. Indeed, confidentiality processes is a known challenge to collaborative work [27], particularly given differences in implementation and regulation of HIPAA and FERPA [12]. Policy implications include recommendations around making consent between schools and PCP offices standard practice at the start of a new school year or at an annual well child check to facilitate collaboration and support youth in need sooner. Feedback from participants guided improvement with communication processes so that a follow-up is sent by email after each virtual huddle to ensure clear communication with point people identified to follow up on next steps. Additionally, as challenges with family follow through on recommendations can be barriers to effective care, identifying point people affiliated with the family or school such as community agency case managers to serve as liaisons can be critical to effective follow through.

Study limitations include small sample size, absence of standardized measurements for participants in collaborative process or students, as well as lack of comparison control group. Additionally, the response rate of 44% is not atypical but may have biased results favorably. The tiered model of collaboration depends on the ability to have liaisons with school teams such as the grant-supported BOCCES team and access to mental health specialists such as through a CPAP type program. This may not be available in all settings, although a strength of this study was demonstrating that even in more rural settings with a shortage of mental health providers, creative telehealth solutions may be helpful. Future studies are recommended to build upon these results and evaluate more rigorously the effectiveness of collaborative huddles on student level outcomes as well as outcomes for PCPs involved in this process compared with those who are not.

## 5. Conclusions

Multidisciplinary school-focused virtual collaborations described in this pilot intervention include critical components of collaborative care, such as psychoeducation through the multidisciplinary process, support and education to PCPs, psychiatric consultation to PCPs through free consultative services, and care coordination with the support of CPAP liaison coordinators and school-based teams. Including trainees in these huddles provides important educational opportunities and helps prepare future healthcare providers to engage in multidisciplinary teams and to experience the potential impact and value of school-focused collaborative models of care. In the absence of integrated school-based health centers and to meet growing mental health needs, creative collaborative models supported by CPAPs can provide feasible avenues to increase communication and coordination between PCPs, school-based teams and mental health providers and has the potential to reduce known youth mental health disparities. Future research is recommended to build upon these findings and more rigorously explore the potential impact of these hybrid models on student outcomes and mental health disparities.

## Figures and Tables

**Table 1:** Demographic information of Tier 5 youth presented at collaborative huddle.

	Male (*n* = 14)	Female (*n* = 5)	Total (N = 19)
*Racial Identity*			
Asian/Pacific Islander	3	0	3
Black/African-American	3	0	3
White	6	5	11
Multiracial	2	0	2
*Ethnicity*			
Hispanic	2	0	2
Non-Hispanic	7	4	11
Unknown	5	1	6
*School Level*			
Elementary School	9	3	12
Middle School	2	1	3
High School	3	1	4

**Table 2:** Case complexity and level of family functioning of Tier 5 youth.

	Male (*n* = 14)	Female (*n* = 5)	Total (N = 19)
*Complexity*			
Simple	0	1	1
Somewhat complex	3	0	3
Complex	10	4	14
Very Complex	1	0	1
*Family Functioning*			
Healthy	1	0	1
Mild-Moderate Dysfunction	6	3	9
Severe Dysfunction	6	2	8
Extreme Dysfunction	1	0	1

**Table 3:** Themes identified from feedback survey and theme prevalence.

*Intervention Impact*	%
Invaluable learning and advice	21%
Multidisciplinary collaboration	17%
Medical or objective lens validating concerns	14%
Improved transitions of care	3%
*Implementation Challenges*	
Follow-up communication and sharing of documentation	10%
Support for family follow up	3%
Extensive paperwork	3%

## Data Availability

The original contributions presented in this study are included in the article. Further inquiries can be directed to the corresponding author.
